# P31 Navigating complex infections in systemic lupus erythematosus: a lupus flare complicated with neutropenic sepsis and a rare fungal infection by Magnusiomyces clavatus

**DOI:** 10.1093/rap/rkae117.062

**Published:** 2024-11-05

**Authors:** Dhanushka Jayamali Withanage, Samanthi Welikumbura, Subhra Raghuvanshi

**Affiliations:** Department of Rheumatology, Wrexham Maelor Hospital, Betsi Cadwaladr University Health Board, Wrexham, United Kingdom; Department of Microbiology, Wrexham Maelor Hospital, Public Health Wales., Wrexham, United Kingdom; Department of Rheumatology, Wrexham Maelor Hospital, Betsi Cadwaladr University Health Board, Wrexham, United Kingdom

## Abstract

**Introduction:**

Systemic lupus erythematosus (SLE) can predispose patients to infections due to active disease or immunosuppressive treatments. Neutropenic sepsis, which could be caused by either, is a life-threatening condition with a challenging management and can be complicated by opportunistic fungal infections. Here we present a case of a lupus flare complicated by neutropenic sepsis and streptococcal pneumonia followed by a cavitary lung lesion and a rare fungal infection by Magnusiomyces clavatus (formally known as Saphrochaete calavata or Geotrichum calavatum). To our knowledge this is the first reported case of Magnusiomyces clavatus infection in a SLE patient in the United Kingdom.

**Case description:**

A 32-year-old Caucasian female with a 6-year history of SLE presented 8 weeks post-partum with fever, cough, oral ulcers and joint pains for 2 weeks. She had a lupus flare in her third trimester, and was on prednisolone tapering dose at 10 mg/day, hydroxychloroquine and azathioprine. Her lupus manifestations were limited to mucocutaneus and musculoskeletal manifestations in the past and never had significant renal or neuropsychiatric involvement.

On physical examination, she was febrile, ill and confused. She had oral ulcers and active synovitis. She was dyspneic with a saturation of 93% and was tachycardic. There were right upper zone crepitations on auscultation. Investigations revealed profound pancytopenia (WBC 0.1 x 10^9/L, neutrophils 0, hemoglobin 8.9 g/dL, platelets 79 x 10^9/L), raised dsDNA titer (219 IU/ml) and low serum complements. Streptococcus pneumoniae was isolated from blood cultures and CT chest showed dense right upper lobe consolidation.

She was diagnosed with lupus flare with neutropenic sepsis and streptococcal pneumonia and managed with intravenous antibiotics, granulocyte colony-stimulating factor (G-CSF), intravenous immunoglobulin, steroids and blood product transfusions. Despite a prolong period of persistent neutropenia, she had a good response to treatment initially. However, after 2 weeks in to her hospital admission her cough worsened with brownish sputum and chest imaging reveled development of a cavitary lesion in the previously consolidated area. Blood cultures isolated Magnusiomyces clavatus fungus, and her beta-D-glucan levels were markedly elevated. However, the bronchoalveolar lavage was negative for the fungus. Notably, she had athletes’ foot which is a known entry for this fungus.

She was treated with intravenous amphotericin B, followed by a prolong course of oral voriconazole. She gradually recovered but remains under evaluation for possible surgical intervention due to the persistent lung cavity.

**Discussion:**

This case highlights the complex interplay between autoimmune disease and infections in patients with SLE and emphasize the management challenges seen in lupus flares complicated with severe infections. The presentation of this patient with neutropenic sepsis and streptococcal pneumonia illustrates the increased risk of infections due to disease activity and immunosuppressive therapy in SLE.

The development of a lung cavity further complicated the disease course and prompted to investigate for opportunistic fungal infections such as aspergillus. Isolation of Magnusiomyces clavatus from blood highlights the need for increased vigilance for rare fungal infections in immunosuppressed patients. Magnusiomyces clavatus is a yeast like fungus and is a rare cause of invasive fungal infections. It is primarily reported in patient with haematological malignancy on chemotherapy and is acquired through environmental exposure. Commonest portals of entry are through respiratory tract, gastrointestinal tract and damaged skin such as athletes foot, which was seen in our patient. It also presents a significant management challenge due to rarity of its occurrence and limited clinical experience and caries high mortality.

This patient’s treatment plan was initially tailored to treat neutropenic sepsis, pneumonia and active lupus. Upon identification of the fungus this was adjusted appropriately to treat the fungaemia with IV amphotericin and oral voriconazole, reflecting the necessity for aggressive therapy for invasive fungal infections. Continues input from microbiology, respiratory and rheumatology teams were taken emphasizing the importance of multidisciplinary approach in managing such challenging cases.

This case highlights importance of early detection and aggressive management of infections in SLE patients. Vigilance for rare opportunistic infections in immunocompromised SLE patients will aid in improving their overall outcome. This case also draws attention to Magnusiomyces clavatus fungal infection, primarily reported in patients with haematological malignancy on chemotherapy, this time observed in a patient with SLE.

**Key learning points:**

• **SLE patients have an increased susceptibility to infections:** Patients with systemic lupus erythematosus (SLE) are at a high risk of infections due to both the disease activity and the immunosuppressive treatment being used. This case highlights the importance of early detection and aggressive treatment of infections in SLE patients.

• **It is challenging to manage neutropenic sepsis in a patient with a concurrent lupus flare:** Neutropenic sepsis can lead to life-threatening complications. The management consists of timely initiation of broad-spectrum antibiotics, granulocyte colony-stimulating factor (G-CSF), all the while treating for a lupus flare as well. It is important to have a fine balance between immunosuppression for lupus flare vs treatment of infection.

• **Immunosuppressed SLE patients, especially when there is severe neutropenia, can develop rare invasive fungal infections:** Magnusiomyces clavatus infections are rare infections and seen mainly in patients with haematological malignancies on chemotherapy. Severe neutropenia that can develop in these patients is a known risk factor for this infection. This case highlights the importance of considering rare fungal infections in immunosuppressed SLE patients with neutropenic sepsis. There should be a high index of suspicion, specially when there is a deterioration of the clinical state despite appropriate treatment for neutropenic sepsis.

• **A multidisciplinary approach is crucial to success when managing SLE patients with complex infections:** Multidisciplinary input from infectious disease specialists, microbiologists, and respiratory physicians when indicated are paramount in optimizing patient outcomes.

